# Antifungal effects of Metformin against *Candida albicans* by autophagy regulation

**DOI:** 10.71150/jm.2411008

**Published:** 2025-04-29

**Authors:** Xiao Zhao, Yang Wang, Qinqin Zhang, Yun Huang, Xin Wei, Daming Wu

**Affiliations:** 1 Department of Endodontics, The Affiliated Stomatological Hospital of Nanjing Medical University, Nanjing 210000, P. R. China; 2 Jiangsu Province Engineering Research Center of Stomatological Translational Medicine, Nanjing 210000, P. R. China; 3 State Key Laboratory Cultivation Base of Research, Prevention and Treatment for Oral Diseases, Nanjing 210000, P. R. China

**Keywords:** *Candida albicans*, metformin, autophagy, antifungal activity

## Abstract

*Candida albicans* (*C. albicans*) is a common opportunistic fungal pathogen that can cause infections ranging from superficial to severe systemic diseases. This study investigates the antifungal effects of metformin on *C. albicans* and explores its underlying mechanisms. Growth inhibition was assessed via XTT assays, and hyphal formation and morphological changes were observed by light microscope and scanning electron microscopy (SEM). Mitochondrial membrane potential (MMP) and reactive oxygen species (ROS) levels were measured with JC-1 and DCFH-DA probes, respectively. Gene expression related to ROS and autophagy was quantified by RT-*q*PCR, and autophagosomes were visualized using transmission electron microscopy (TEM). Metformin significantly inhibited *C. albicans* growth and hyphal formation, altered cell morphology, reduced MMP, and increased ROS levels. It activated autophagy in planktonic *C. albicans* but suppressed it in biofilm forms. Additionally, metformin exhibited synergistic effects with amphotericin B against planktonic *C. albicans* and with caspofungin against biofilms. The findings suggest that metformin exerts antifungal activity by modulating MMP, ROS levels, and autophagy-related pathways, and enhances the efficacy of specific antifungal drugs.

## Introduction

*Candida albicans* (*C. albicans*) is a major opportunistic fungal pathogen in humans, commonly residing in the oral cavity, gastrointestinal tract, and vagina (Luther et al., 2023). While it often causes superficial infections, *C. albicans* poses significant risks to immunocompromised individuals, including patients with AIDS, those undergoing chemotherapy, or organ transplant recipients, resulting in high morbidity and mortality rates (d'Enfert et al., 2021; Koshikawa et al., 2022). Additionally, *C. albicans* has the ability to form biofilms on host tissues and medical devices surfaces. These biofilms consist of yeast, pseudo mycelium, and hyphal cells embedded in an extracellular matrix, exhibiting a highly organized structure (Osset-Trénor et al., 2023; Pereira et al., 2021). Biofilms confer enhanced resistance to environmental stressors and antifungal agents, exhibiting resistance levels significantly higher than those required to target planktonic cells (Mathé and Van Dijck, 2013; Rabaan et al., 2023). This heightened resistance poses a significant challenge for traditional antifungal treatments, particularly in eradicating *Candida* biofilms.

The development of novel antifungal drugs is arduous for difficulties related to toxicity, pharmacokinetic complexities, and high costs. To address these obstacles, drug repurposing was proposed, which identifies new use for approved or developing drugs that are outside the scope of the original medical indication (Moraes and Ferreira-Pereira, 2019). Several non-antifungal drugs have demonstrated antifungal activity or enhanced the efficacy of antifungal drugs when used in combination (Barbarossa et al., 2023; Lin et al., 2023; Moraes and Ferreira-Pereira, 2019). Metformin is an oral medication commonly used for the treatment of type 2 diabetes (Podhorecka et al., 2017). Recent studies reveal that metformin not only affects metabolic pathways but also influences cellular autophagy levels by modulating related signaling pathways such as AMPK/mTOR (AMP-activated protein kinase / mechanistic target of rapamycin) (Bridges et al., 2014; González et al., 2020; Lv and Guo, 2020). Autophagy, a cellular mechanism for degradation and resource recycling, is widely conserved among eukaryotes (Miceli et al., 2023). In microorganisms, it acts as a vital stress response, helping cell adapt to challenging conditions like nutrient deprivation and oxidative stress (Bekbulat, 2023). Studies indicate that metformin, under specific conditions such as hypoxia or in cancer cells, can inhibit autophagy by modifying the intracellular metabolic state or interacting with other signaling pathways (Lee et al., 2023; Lu et al., 2021).

*C. albicans* utilize autophagy to adapt to unfavorable conditions, such as nutrient scarcity and oxidative stress (Du et al., 2022; Wójcik-Mieszawska et al., 2023). Through this process, *C. albicans* regulates intracellular nutrient acquisition and maintains metabolic balance, enabling survival under stressful conditions (Du et al., 2023). Previous studies have shown that antifungal agents can influence *C. albicans* autophagy (Shen et al., 2023). However, whether metformin affects *C. albicans* by modulating autophagy remains unclear. Research has demonstrated that metformin enhances the susceptibility of tetracycline-resistant *E. coli* to tetracycline (Liu et al., 2020), and exhibits antifungal activity against *C. glabrata* in combination with voriconazole, fluconazole, and amphotericin (Xu et al., 2018). However, no studies have yet explored the effects and mechanisms of metformin on *C. albicans*. This study aims to investigate the effects of metformin on the growth of *C. albicans* in both planktonic and biofilm states, examine its potential antifungal mechanism through autophagy modulation, and evaluate its efficacy when combined with other antifungal drugs.

## Materials and Methods

### Experimental strains

The *C. albicans* standard strain (SC5314, ATCC VR MYA2876^TM^) was purchased from the American Type Culture Collection (ATCC, USA) and stored at -80℃ until needed. Yeast cells were subcultured on Sabouraud dextrose agar (SDA, Hope Bio-Technology Co., Ltd., China) plates, and a fresh single colony was selected and inoculated into YPD liquid medium (Sigma, USA). The culture was incubated overnight at 30°C with shaking at 210 rpm. A 1 ml fungal suspension was collected in a 1.5 ml Eppendorf tube, centrifuged at 2,100 × g for 5 min, and the supernatant discarded. The pellet was washed thrice with PBS and resuspended in PBS to obtain the *C. albicans* suspension.

### Drugs

All drugs (metformin, fluconazole, itraconazole, amphotericin B, caspofungin, 5-fluorocytosine, and terbinafine) were procured from Sigma. Drug solutions were prepared according to Clinical and Laboratory Standards Institute guidelines (CLSI, 2017). Metformin, fluconazole, caspofungin, and 5-fluorocytosine were dissolved in sterile distilled water to achieve the required concentrations. Itraconazole, amphotericin B, and terbinafine were prepared in dimethyl sulfoxide (DMSO) (Sigma, USA). All solutions were stored at -20°C until use.

### Minimal inhibitory concentration 80% (IC_80_) of metformin against planktonic *C. albicans*

The IC_80_ refers to the lowest concentration of a compound required to inhibit 80% of microbial growth under specific conditions. In this study, the IC_80_ of metformin against planktonic *C. albicans* was determined using broth microdilution and XTT assays (Chen et al., 2024). Briefly, *C. albicans* suspension was diluted to 2 × 10^3^ CFU/ml with RPMI-1640 medium (Sigma, USA), adjusted to pH 7.0 using MOPS buffer (Sigma, USA). A 100 μl of this suspension and add it to a 96-well plate containing 100 μl of serial 2-fold diluted metformin, with concentrations from 0. 5 to 128 mg/ml, and set control wells (the biofilm without metformin). After incubation at 37℃ for 24 h in a CO_2_ incubator (ESCO, Singapore), 100 μl of the suspension was transferred to a new 96-well plate. Then, 100 μl of XTT solution (Invitrogen, USA) was added, and the plate was incubated in the dark for 2 h. Absorbance was measured at 492 nm using a microplate reader, and the IC_80_ was calculated.

### Sessile minimal inhibitory concentration 50% (IC_50_ of biofilm formation) of metformin against *C. albicans* biofilm

The method for determining the IC_50_ of metformin against mature biofilms was similar to that for IC_80_. Briefly, *C. albicans* suspension was adjusted to 1.0 × 10^6^ CFU/ml and seeded into 96-well plates, followed by incubation at 37℃. After 2 h, the medium was aspirated, and the wells were washed thrice with sterile PBS to remove non-adherent fungi. The fungi were then incubated for an additional 24 h. 100 μl serial 2-fold dilutions of the metformin, ranging in concentration from 0. 5 to 128 mg/ml, were dispensed into the 96-well plates, while the control biofilms without metformin were established accordingly. Following incubation at 37℃ for another 24 h, the susceptibilities of the biofilms were assessed using the XTT assay. Biofilm growth was quantified by measuring absorbance at 492 nm using a microplate reader and calculate IC_50_ of biofilm formation. All experiments were conducted thrice.

### Time-kill curve assay of metformin against *C. albicans*

To examine the effects of concentration and exposure duration, a time-kill assay was performed to assess the antifungal effects of metformin on *C. albicans* in planktonic and biofilm (Zhou et al., 2012). Briefly, *C. albicans* suspension was diluted to 1 × 10^3^ CFU/ml in RPMI 1640 medium with metformin (1/2 IC_80_, IC_80_, 2 IC_80_, and 4 IC_80_) in 96-well plates. As described above, mature biofilms were cultivated in 96-well plates. Following this, the medium was removed, and biofilms were washed twice with PBS. Subsequently, 100 μl of metformin at concentrations equivalent to 1/2 IC_50_, IC_50_, 2 IC_50_, and 4 IC_50_ of biofilm formation was added to the RPMI 1640 medium. The control biofilms without metformin were set up accordingly. During the experiment, all samples were incubated at 37°C in a CO_2_ incubator. At designated time points (0, 6, 12, 18, 24, 30, 36, 42, 48, 54, 60, 66, and 72 h), the XTT assay was used to assess fungal susceptibility, as described above.

### Hyphae growth assay of *C. albicans*

To assess the effect of metformin on hyphal growth, RPMI 1640 medium, which induces hyphae formation, was used. *C. albicans* (5.0 × 10^5^ CFU/ml) were suspended in medium supplemented with metformin concentrations of 8, 16, 32, 64, and 128 mg/ml, then plated onto 24-well tissue culture plates. Following a 3 h incubation at 37℃ in a CO_2_ incubator, cell morphology was captured using the light microscope. Then the mycelial inhibition rate was calculated by measuring mycelial length.

### Scanning electron microscope (SEM)

After co-culture with different concentrations of metformin, *C. albicans* in planktonic and biofilm were washed thrice with PBS and fixed in 2.5% glutaraldehyde overnight at 4℃. The samples were dehydrated in different concentrations of ethanol (30, 50, 70, 80, 90, and 100%) gradient, with each step lasting 15 min, followed by freeze-drying. Subsequently, the dried samples were affixed to conductive adhesive, mounted onto copper sample holders, and sputter-coated with gold under vacuum conditions. Morphological observations were performed using SEM (LEO, Germany).

### Mitochondrial membrane potential (MMP) measurement

To evaluate changes in MMP, the fluorescent probe JC-1 were used. Planktonic *C. albicans* were adjusted to 1 × 10^3^ CFU/ml, and mature biofilm were adjusted to 1 × 10^6^ CFU/ml. Both were treated with different concentrations of metformin at 37˚C for 4 h. Subsequently, the cells were stained with 10 μmol/L JC-1 at 37˚C for 30 min in the dark according to instructions. Fluorescence measurements were performed for JC-1 monomers (excitation: 514 nm, emission: 529 nm) and JC-1 polymers (excitation: 585 nm, emission: 590 nm). The ratio of fluorescence intensities was calculated to assess changes in MMP.

### ROS level detection

ROS levels were determined using fluorescence microscopy and the probe 2',7'-Dichlorodihydrofluorescein diacetate (DCFH-DA). For planktonic *C. albicans*, cells were diluted to 1 × 10^6^ CFU/ml in medium and exposed to varying metformin concentrations at 37℃ for 4 h. For *C. albicans* biofilm, mature biofilms cultured on 96-well plates were treated with metformin under the same conditions. Negative controls were incubated without metformin at the same condition. After incubation, all groups were washed twice with PBS buffer and stained with 10 μmol/L DCFH-DA at 37℃ for 30 min in the dark. Then, they were washed thrice with PBS, and read relative fluorescence intensity values with a fluorescence microplate reader.

### RT-*q*PCR

The expression of ROS related genes (*trr1*, *sod1-5*, *cat1-2*, and *glr1*) and autophagy related genes (*atg1-10*, *atg12-13*, *atg16-17*, *atg27*, and *ccz1*) were evaluated by the quantitative Reverse Transcription Polymerase Chain Reaction (RT-*q*PCR). Both planktonic and biofilm states of *C. albicans* were treated with varying concentrations of metformin. Total RNA was extracted with Trizol reagent (Invitrogen, USA), followed by cDNA synthesis using a kit (TaKaRa Bio, China) as per the manufacturer’s instructions. The resulting cDNA was stored at -80°C until required. Subsequently, real-time PCR analysis was performed on an ABI 7300 Fast Real-time PCR machine (Applied Biosystems, Switzerland) using Absolute *q*PCR SYBR Green Mix (Thermo Scientific, USA). The PCR amplification protocol consisted of an initial denaturation step at 95℃ for 10 min, followed by 40 cycles of denaturation at 95℃ for 15 s, annealing at 55℃ for 60 s, and extension at 72℃ for 20 s. After amplification, a melting curve analysis was conducted to confirm the absence of primer dimers. Gene expression levels were quantified using the 2^-ΔΔCt^ method with *act1* serving as the internal reference gene (Yu et al., 2011). Primer design was outsourced to Shanghai Generay BioTech Co., Ltd. The primers and sequences are listed in Tables 1 and 2. The experiment was conducted thrice.

### Transmission electron microscopy (TEM) observation

After co-culture with metformin, *C. albicans* cells in planktonic and biofilm were washed thrice with PBS and placed in 2.5% glutaraldehyde overnight at 4℃. Subsequently, the fungi, both planktonic and biofilm, were treated with 1% osmium tetroxide for 2 h at 4℃. Sequentially, ethanol dehydrated steps (30, 50, 70, 80, 95, and 100%) were performed, followed by embedding the cells in epoxy resins, sectioning, and staining. Finally, the samples were examined using TEM (JEM-2100, JEOL, Japan).

### Interaction between metformin and antifungal drugs

The synergistic effects of metformin in combination with antifungal drugs (fluconazole, itraconazole, caspofungin, amphotericin B, terbinafine, and 5-flucytosine) were evaluated using a microdilution checkerboard technique and XTT assay. For planktonic *C. albicans*, 50 μl of a 2-fold concentration of antifungal drugs was added to columns 1–11, while 50 μl of a 2-fold concentration of metformin was added to rows A–G of microtiter plates, and 100 μl solution with final yeast concentrations of 1 × 10^3^ CFU/ml. Final drug concentrations ranged from 0.0315 to 16 μg/ml for fluconazole, itraconazole, amphotericin B, and 5-flucytosine; from 0.25 to 128 μg/ml for terbinafine; and 0.5 to 128 mg/ml for metformin. Two control groups (biofilm with and without metformin) and blank wells (no biofilm or drug) were included. Plates were incubated at 37°C for 24 h. Survival rates were assessed using XTT assays. For the *C. albicans* biofilm, mature biofilms in 96-well plates were prepared as previously described. After aspiration of the medium and washing of biofilms with PBS, 50 μl of a 2-fold concentration of antifungal drugs was added to columns 1–11, and 50 μl of a 2-fold concentration of metformin was added to rows A–G. Antifungal drug concentrations ranged from 0.0315 to 16 μg/ml for itraconazole and amphotericin B, from 0.125 to 64 μg/ml for fluconazole and 5-fluorocytosine, and from 0.25 to 128 μg/ml for caspofungin and terbinafine. Metformin concentrations were the same as for planktonic *C. albicans*. Following incubation at 37℃ for 24 h, XTT assays were conducted to determine the IC. The fractional inhibitory concentration index (FICI) was used to characterize the synergistic effect between metformin and antifungal drugs against *C. albicans* in planktonic and biofilm (Barbarossa et al., 2024; Sturaro et al., 2024). The FICI was described using the following equation:

FICI=FIC_A_+FIC_B_=C_A_/IC_A_+C_B_/IC_B_

In the formula above, IC_A_ and IC_B_ are respectively the IC_50_ when drug A and drug B act alone, while C_A_ and C_B_ are the IC_50_ when drug A and drug B are used in combination to achieve the same effect. Synergism, additivity, indifference, and antagonism were defined based on FICI values: FICI ≤ 0.5 (synergism), 0.5 < FICI ≤ 1 (additivity), 1 < FICI ≤ 2 (indifference), and FICI > 2 (antagonism).

### Statistical analysis

Statistical analysis was conducted using IBM SPSS statistical software (version 22.0). Results were presented as the mean values ± standard deviation (SD). One-way analysis of variance (ANOVA) followed by Fisher LSD’s post hoc test was employed for data analysis and subsequent comparisons. Statistical significance was set at *p* < 0.05.

## Results

### Antifungal activity of metformin against *C. albicans*

**Fig. 1.**
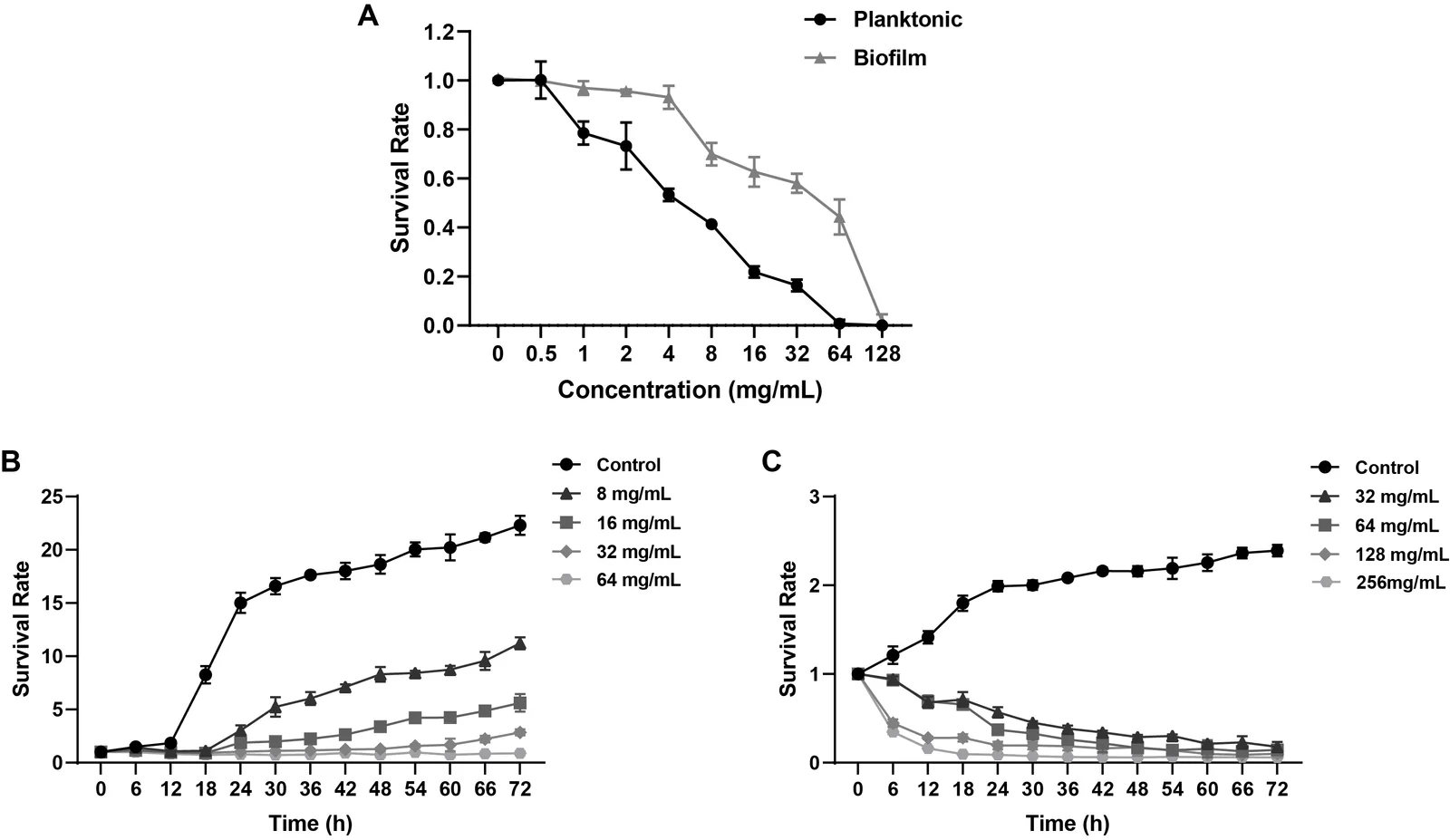
Antifungal activity of metformin against *C. albicans*. (A) Survival rate of *C. albicans* in planktonic and biofilm after treatment with metformin at different concentrations. (B) The time-kill curves of metformin against planktonic *C. albicans*. (C) The time-kill curves of metformin on *C. albicans* biofilms.

Two states of *C. albicans* were treated with different concentrations of metformin. The IC_80_ of metformin inhibiting 80% growth of planktonic *C. albicans* was 16 mg/ml and the IC_50_ of metformin inhibiting 50% growth of *C. albicans* biofilm was 64 mg/ml (Fig. 1A).

Next, in planktonic cells, 1/2 IC_80_, IC_80_, 2 IC_80_, and 4 IC_80_ were used, with concentrations of 8, 16, 32, and 64 mg/ml, respectively. In biofilm, 1/2 IC_50_, IC_50_, 2 IC_50_, and 4 IC_50_ were used, with concentrations of 32, 64, 128, and 256 mg/ml, respectively. In planktonic state, metformin reduced the time-kill curve slope of *C. albicans* compared with the control group, and the greater the concentration, the more significant the reduction was (Fig. 1B). Similarly, in biofilm state, the time-kill curve slope of control group was positive, while that of the metformin against *C. albicans* was negative and decreased with the increase of metformin concentration (Fig. 1C).

### Inhibition by metformin on hyphal development of *C. albicans*

**Fig. 2.**
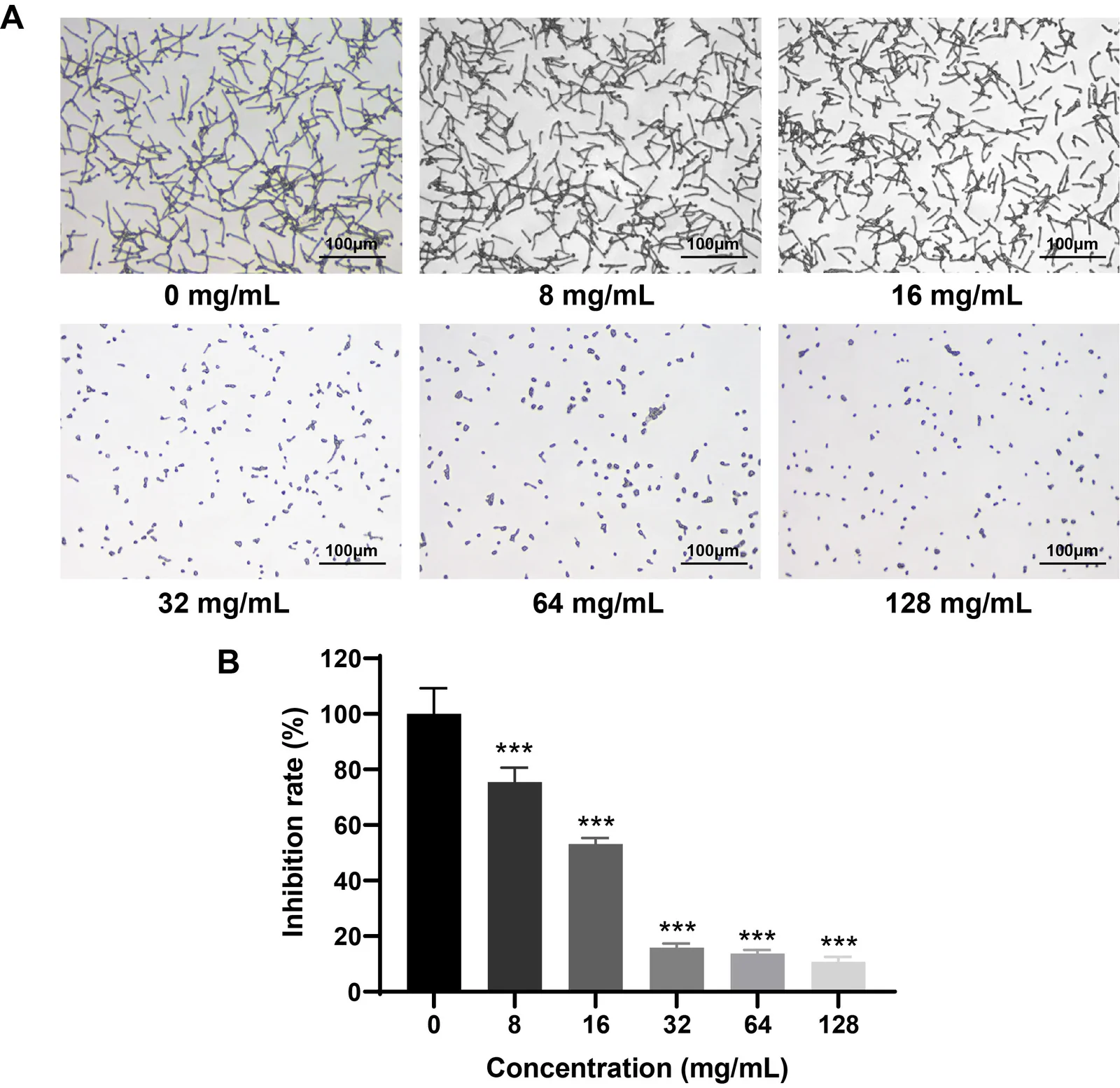
Effects of metformin on hyphal development of *C. albicans*. (A) The images of the hyphal development of *C. albicans* treated by different concentrations of metformin. (B) The hyphal inhibition rate of *C. albicans* after the treatments of different concentrations of metformin. ^***^p < 0.001.

We evaluated the effects of metformin on the hyphal development of *C. albicans*. *C. albicans* developed elongated and regular hyphae without metformin treatment. However, the formation of hyphae was partially inhibited with 8 and 16 mg/ml metformin treatment. Notably, hyphae development was further significantly inhibited after treatment with 32, 64, and 128 mg/ml of metformin (Fig. 2A). The results of hyphae length inhibition rate indicated that metformin impeded the growth of *C. albicans* hyphae (*p* < 0.001), and the inhibition was dose-dependent (Fig. 2B).

### Changes by metformin on the morphology of *C. albicans*

**Fig. 3.**
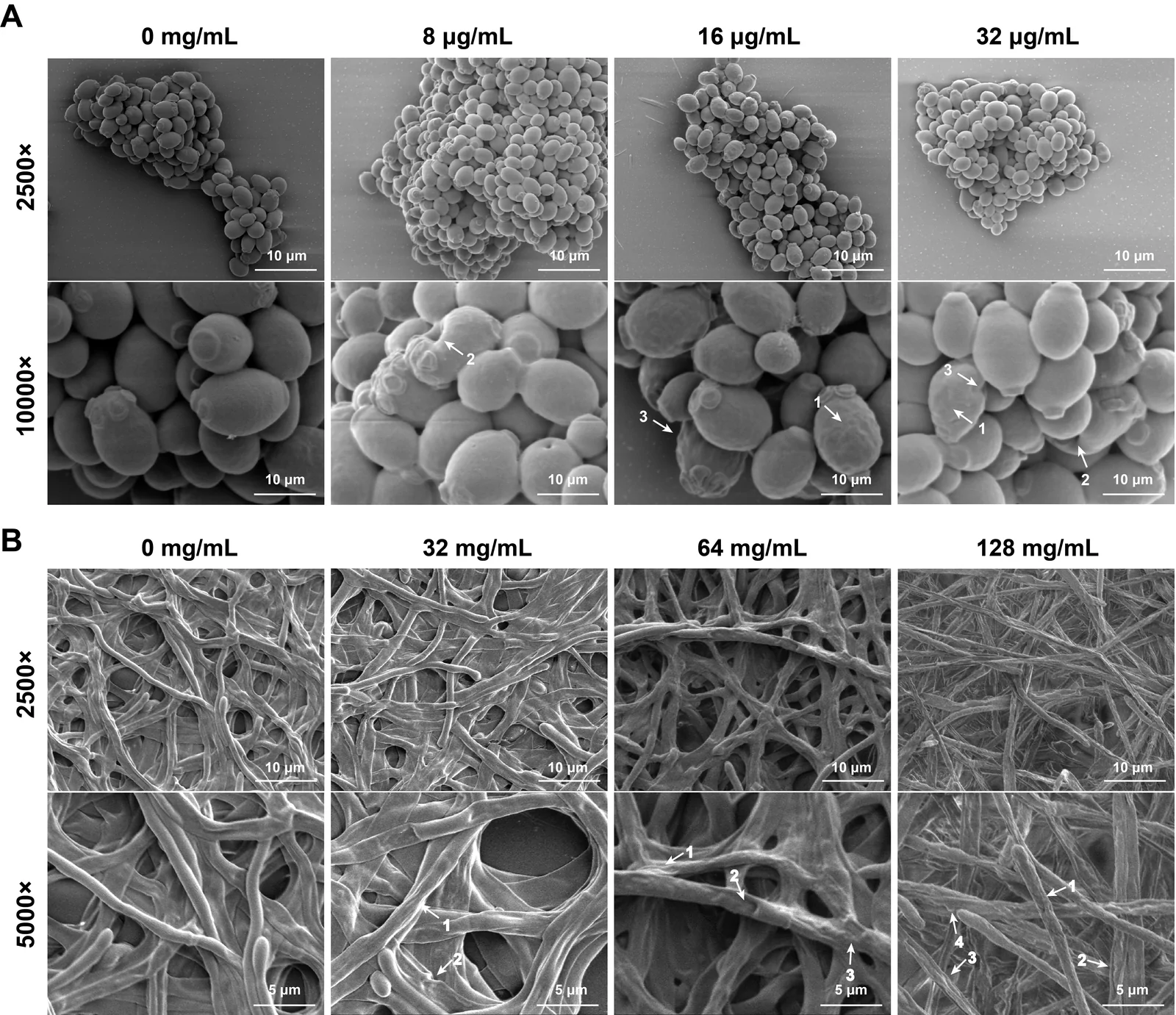
SEM images of *C. albicans* in planktonic and biofilm. (A) Treatment of planktonic *C. albicans* with different concentrations of metformin. (B) Treatment of *C. albicans* biofilms with different concentrations of metformin. Arrows 1 indicates rough surface, 2 indicates collapse, 3 indicates shrinkage, 4 indicates bulge.

For planktonic *C. albicans*, SEM observation showed that compared to the control group, the cell of metformin-treated group appeared rough surfaces, collapsed, and shrinkage (Fig. 3A). For biofilm, SEM observation showed that compared with control group, the surface of biofilm treated with metformin became rough, and the biofilm appeared remarkably morphologically damaged with visible shrinkage, collapse, and bulge (Fig. 3B).

### Effects of metformin on MMP of *C. albicans*

**Fig. 4.**
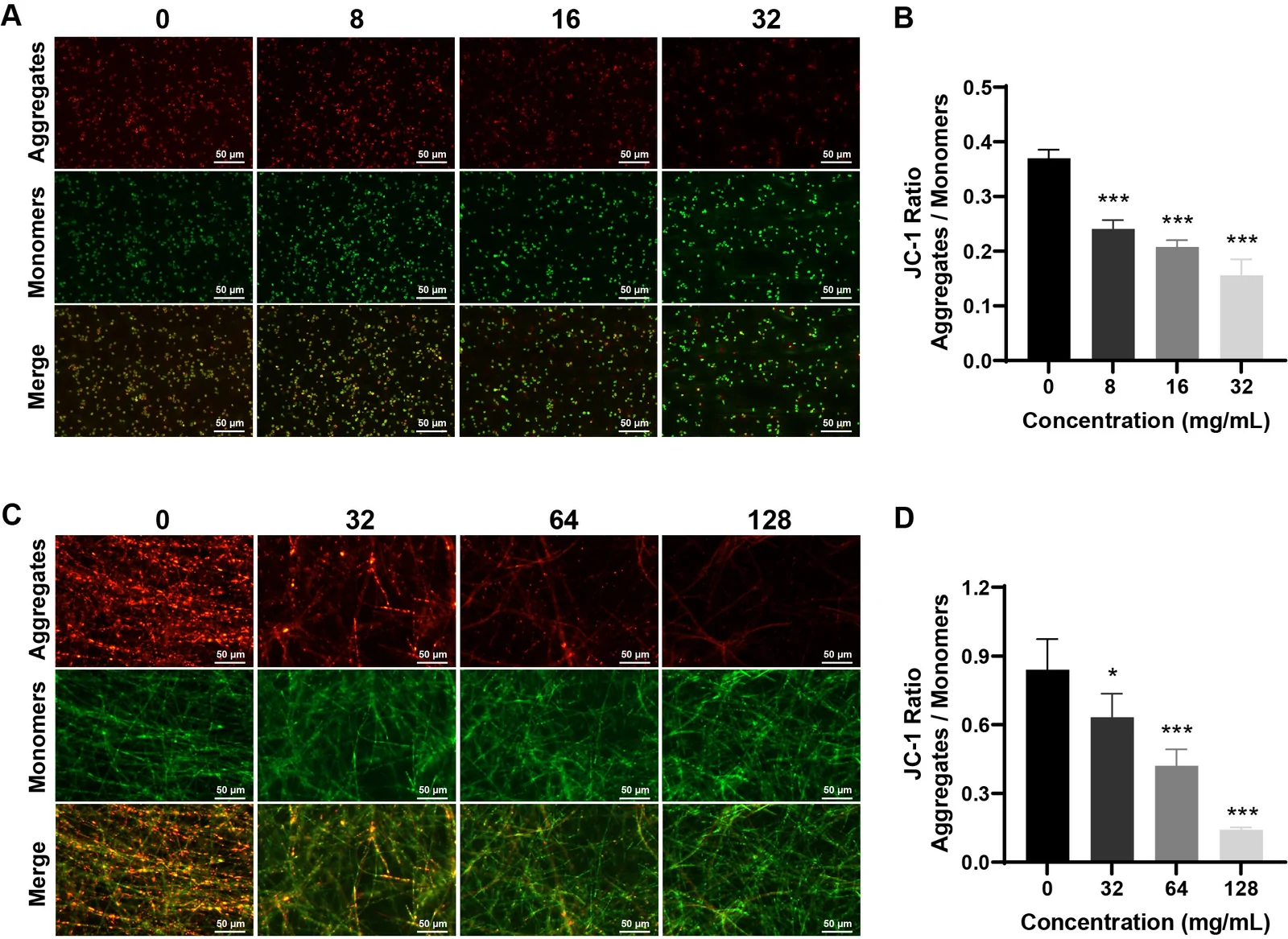
MMP detection of *C. albicans* in planktonic and biofilm. (A, B) Fluorescence images of MMP and ratio of JC-1 aggregates to JC-1 monomers in planktonic *C. albicans* after metformin treatment. (C, D) Fluorescence images of MMP and ratio of JC-1 aggregates to JC-1 monomers in *C. albicans* biofilms after metformin treatment. ^*^p < 0.05, ^***^p < 0.001.

MMP changes in *C. albicans* were determined by the fluorescent probe JC-1. In normal mitochondria, JC-1 forms a JC-1 aggregate in the mitochondria and emits red fluorescence; otherwise, it exists as a JC-1 monomer in the cytosol and emits green fluorescence. The results showed that the red fluorescence of *C. albicans* in planktonic and biofilm was weakened, while the green fluorescence was enhanced after metformin treatment (Fig. 4A and 4C). The ratio of JC-1 aggregates (red fluorescence) to JC-1 monomers (green fluorescence) decreased (*p* < 0.05) in a dose-dependent manner (Fig. 4B and 4D).

### Effects of metformin on ROS levels and related genes expression in *C. albicans*

**Fig. 5.**
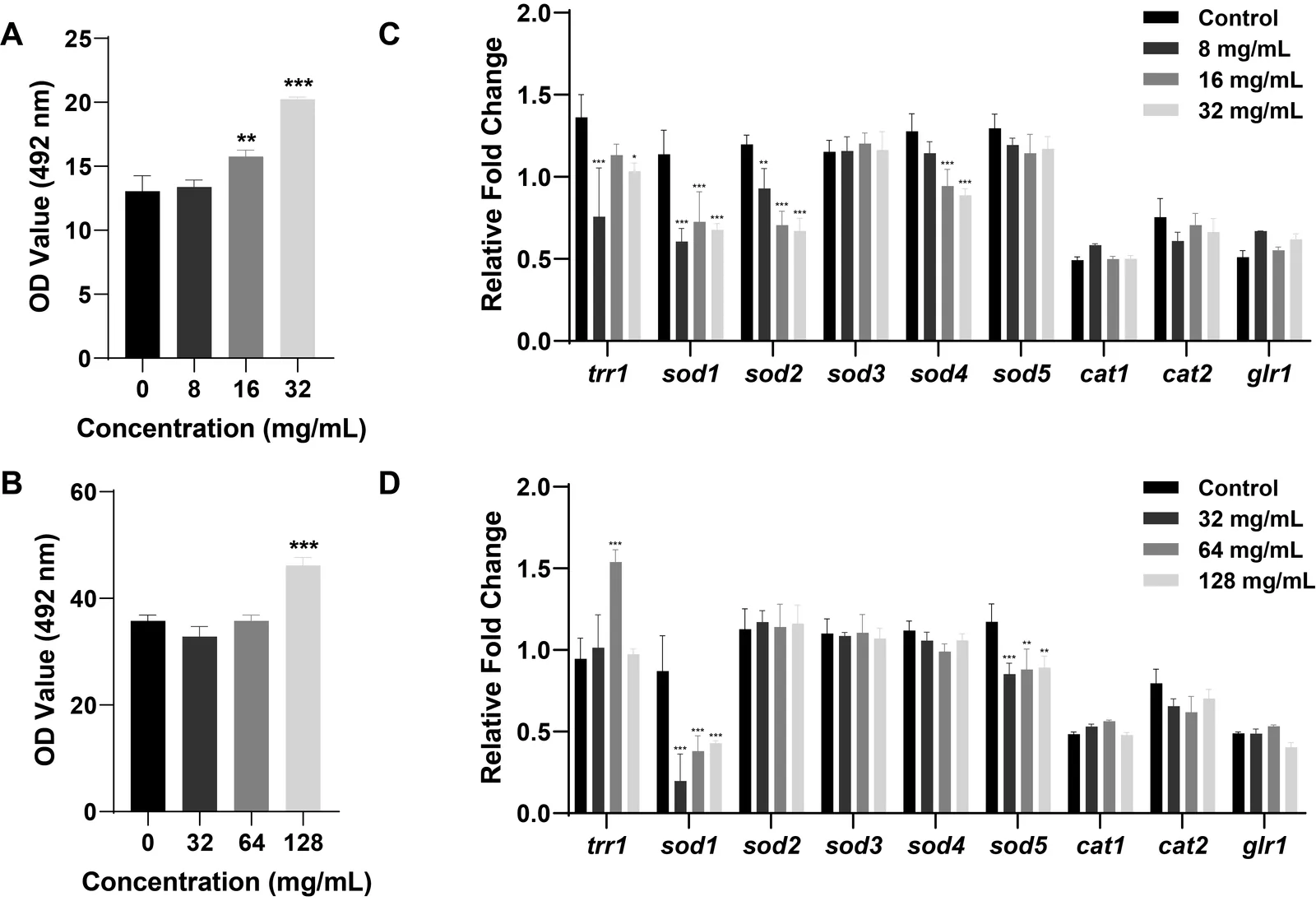
Detection of ROS levels and related genes expression in *C. albicans*. (A) ROS levels in planktonic *C. albicans* and (B) expression levels of antioxidant-related genes. (C) ROS levels in *C. albicans* biofilm and (D) expression levels of antioxidant-related genes. ^*^p < 0.05, ^**^p < 0.01, ^***^p < 0.001.

For planktonic cells, compared to the control group, the 16 and 32 mg/ml metformin-treated groups produced more ROS (*p* < 0.01) (Fig. 5A). In addition, the expressions of some antioxidant-related genes were down-regulated after metformin treatment. In the 8 and 32 mg/ml metformin treatment groups, the gene expression of trr1 was significantly reduced compared to the control group (*p* < 0.05). Similarly, the expressions of *sod1-2* and *sod4* were lower in metformin-treated groups compared to the control group (*p* < 0.001). There were no statistically significant differences in the expression of other genes (Fig. 5B).

For biofilm, compared control group, the 128 mg/ml metformin group produced more ROS (*p* < 0.001) (Fig. 5C). In addition, the expressions of some antioxidant-related genes were up-regulated or down-regulated after metformin treatment. The gene expression of *trr1* in 64 mg/ml metformin-treated group was elevated compared to that in the control group (*p* < 0.001), the gene expressions of *sod1* and *sod5* in 32, 64, and 128 mg/ml metformin-treated groups were lower than that in the control group (*p* < 0.001), and the expression of other genes had no statistical difference (Fig. 5D).

### Autophagy-related genes expression in *C. albicans*

**Fig. 6.**
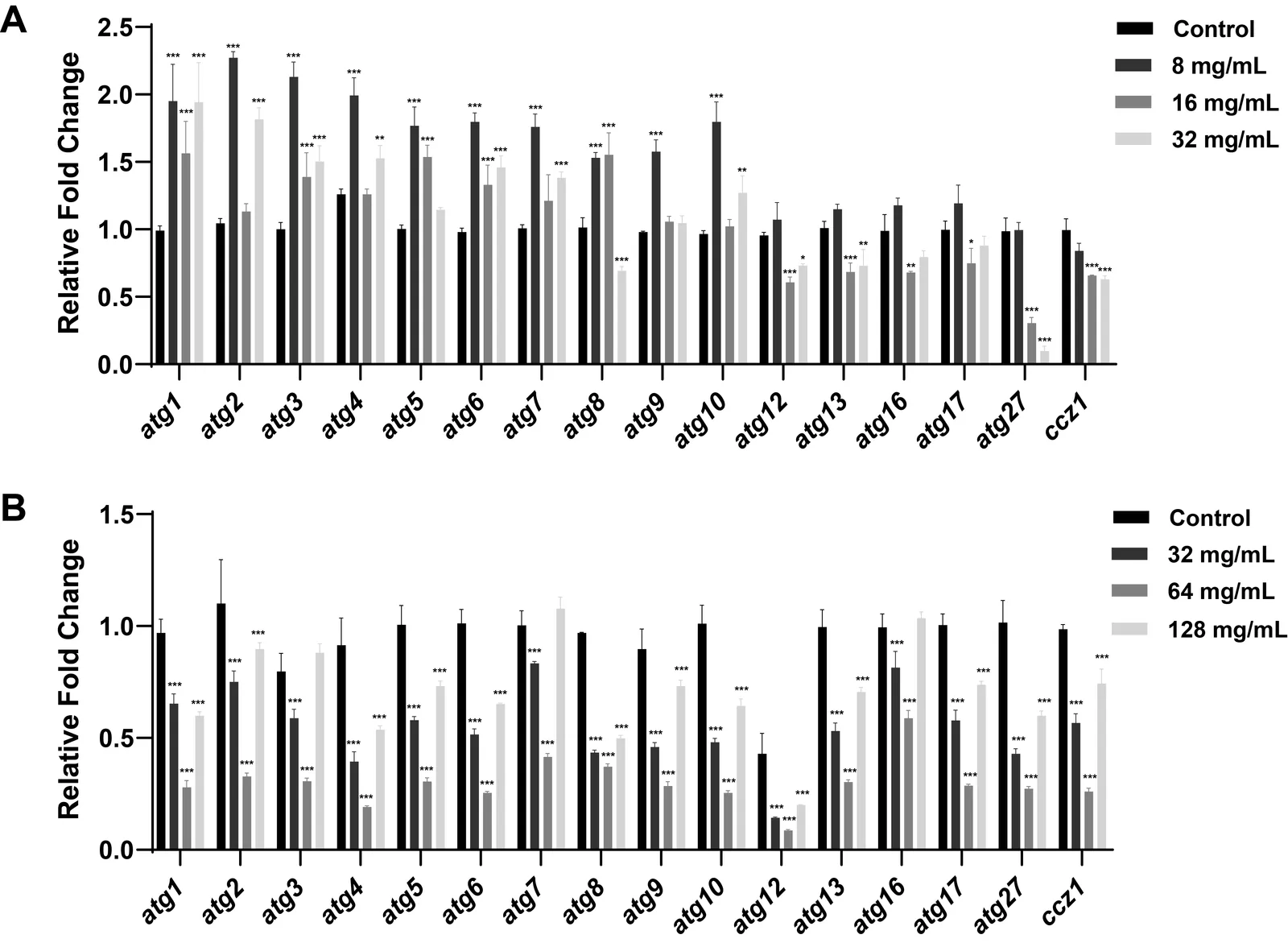
Effects of metformin on autophagy-related genes expression in *C. albicans*. (A) Expression of autophagy related genes in planktonic *C. albicans*. (B) Expression of autophagy related genes in *C. albicans* biofilm. ^*^p < 0.05, ^**^p < 0.01, ^***^p < 0.001.

Figure 6A presented the expression changes of 16 autophagy-related genes in planktonic *C. albicans*. In the 8 mg/ml metformin-treated group, *atg1-10* expressions were up-regulated compared to the control (*p* < 0.001). At 16 mg/ml, *atg1*, *atg3*, *atg5*, *atg6*, and *atg8* were up-regulated (*p* < 0.001), while *atg12-13*, *atg16-17*, *atg27*, and *ccz1* were down-regulated compared to the control (*p* < 0.05). In the 32 mg/ml group, *Atg1-4*, *Atg6-7*, and *Atg10* were up-regulated (*p* < 0.01), while *Atg8*, *atg12-13*, *atg27*, and *ccz1* were down-regulated compared to the control (*p* < 0.05). Figure 6B showed gene expression changes in *C. albicans* biofilms. In the 32 and 64 mg/ml metformin-treated groups, all 16 autophagy-related genes were downregulated compared to the control (*p* < 0.001). At 128 mg/ml, except for *atg3*, Atg7, and *atg1*6, the other 13 genes were downregulated compared to the control (*p* < 0.001).

### Autophagosomes observation in *C. albicans*

**Fig. 7.**
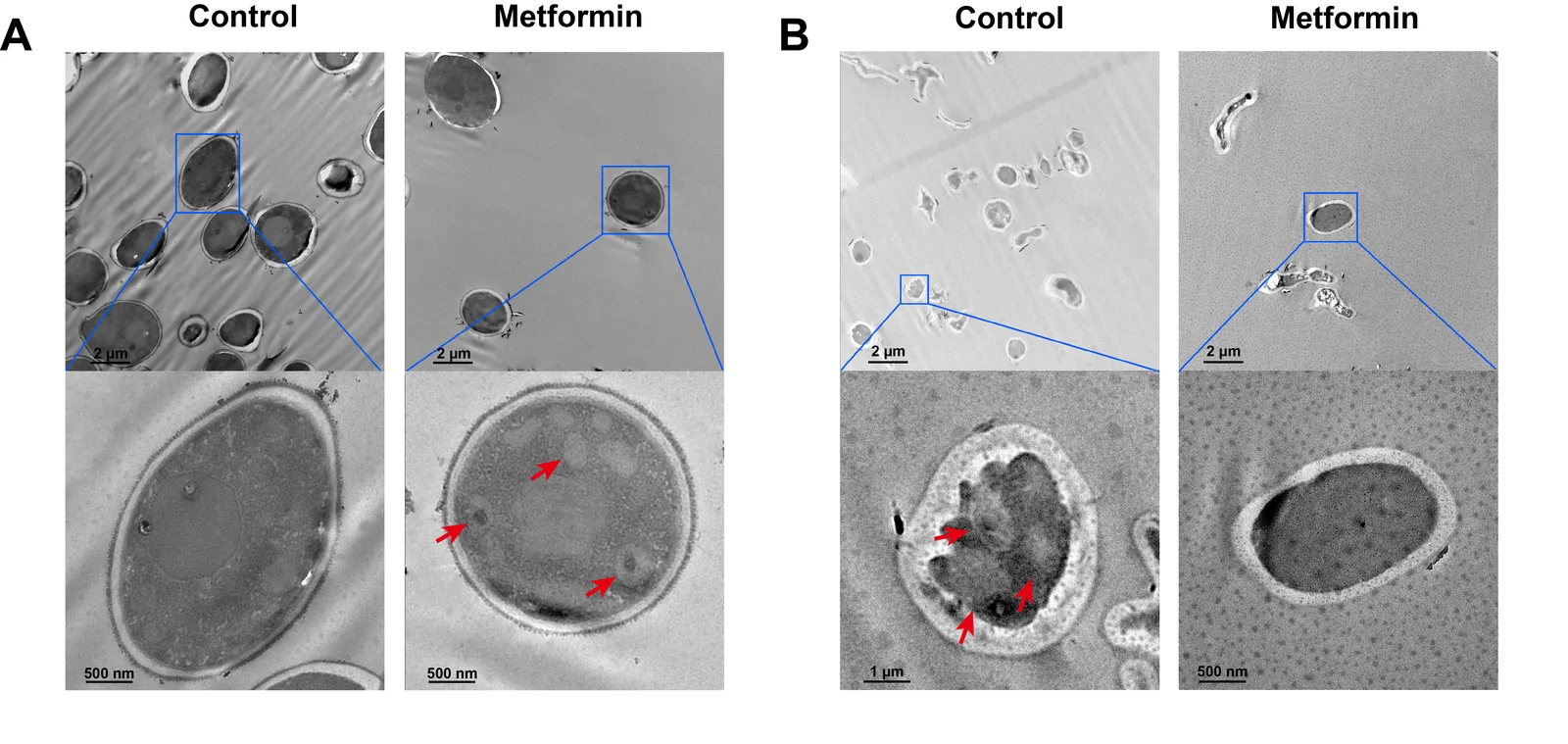
TEM images of *C. albicans* autophagosomes. (A) TEM images of planktonic *C. albicans* in the control group and treated with 16 mg/ml metformin. (B) TEM images of *C. albicans* biofilm in the control group and treated with 64 mg/ml metformin. The red arrow represents autophagosome.

TEM images revealed that planktonic *C. albicans* treated with 16 mg/ml metformin exhibited more double-membrane autophagosomes than the control group, indicating that metformin promoted autophagosome formation in planktonic cells (Fig. 7A). In contrast, *C. albicans* biofilm treated with 64 mg/ml metformin displayed fewer autophagosomes, whereas the untreated biofilm exhibited more autophagosomes (Fig. 7B), suggesting that metformin reduced autophagosome formation in biofilm cells.

### Interactions between metformin and antifungal drugs against *C. albicans* in planktonic and biofilm

**Fig. 8.**
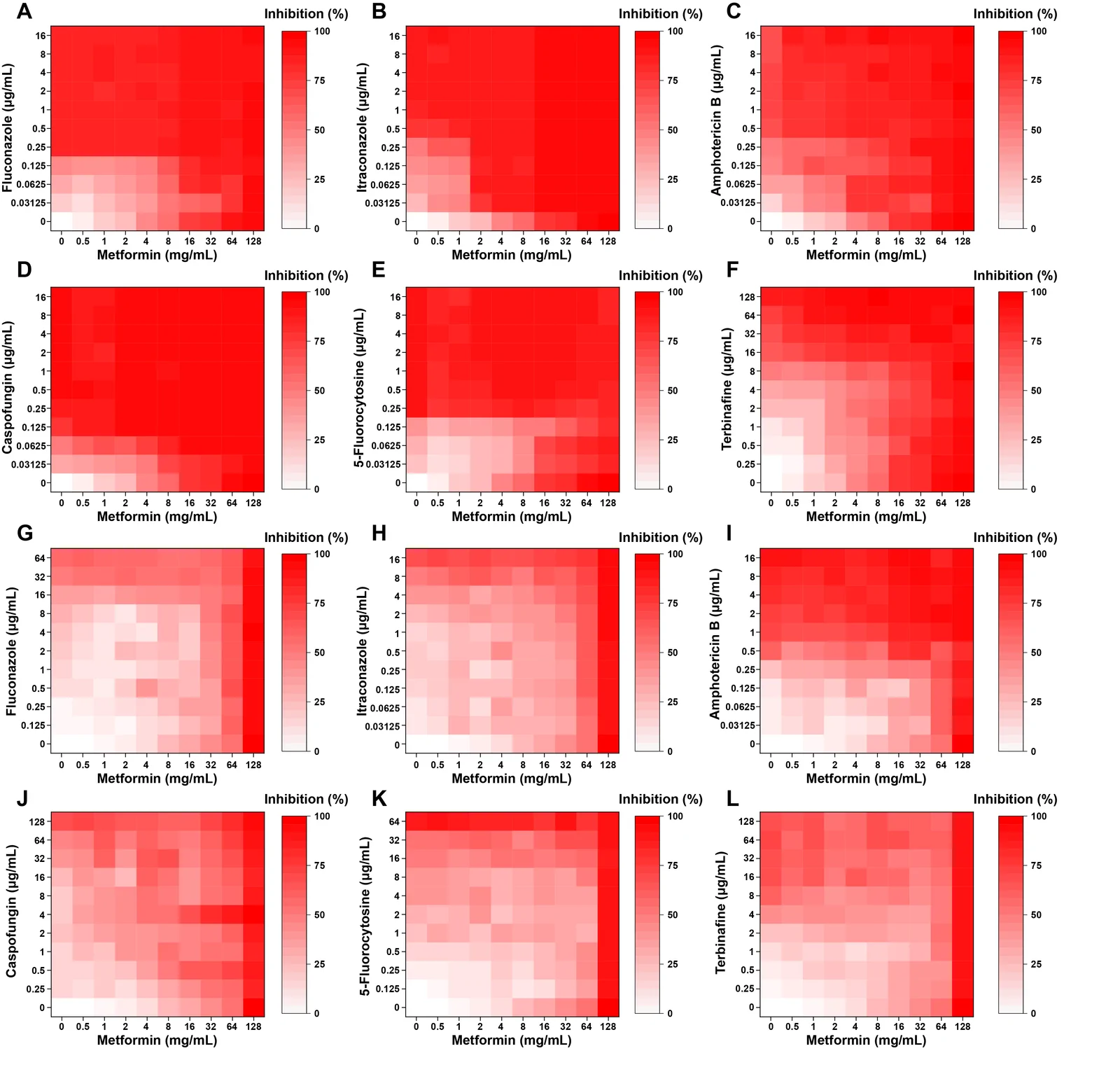
Metformin combined with various antifungal drugs for the treatment of *C. albicans* in planktonic and biofilm. (A–F) Planktonic *C. albicans*, including (A) fluconazole, (B) itraconazole, (C) amphotericin B, (D) caspofungin, (E) 5-fluorocytosine, and (F) terbinafine. (G–L) *C. albicans* Biofilm, including (G) fluconazole, (H) itraconazole, (I) amphotericin B, (J) caspofungin, (K) 5-fluorocytosine, and (L) terbinafine.

For planktonic *C. albicans*, various antifungal drugs combined with metformin showed a synergistic effect when used with amphotericin B and an additive effect with itraconazole. Additionally, fluconazole, caspofungin, and terbinafine in combination with metformin showed indifferent effects, while 5-fluorocytosine exhibited an antagonistic effect when used with metformin (Fig. 8A–F, Table 3). For *C. albicans* biofilm, a synergistic effect existed when caspofungin-combined with metformin, while fluconazole, itraconazole, and amphotericin B had an additive effect when combined with metformin. Furthermore, the combination of 5-fluorocytosine and terbinafine with metformin exhibited indifferent effects (Fig. 8G–L, Table 3).

## Discussion

Increasing the dosage of antifungal drugs to address drug-resistant *C. albicans* infections in clinical practice not only increases side effects but also accelerates *C. albicans* resistance and raises costs (Todd et al., 2023). Drug repositioning and combined use may reduce the drug side effects and cost (Efentakis et al., 2024; Moraes and Ferreira-Pereira, 2019). This study evaluated the antifungal activity of metformin against both planktonic and biofilm states of *C. albicans*, exploring its potential mechanisms of action and effects when combined with antifungal drugs. Biofilms are intricate 3D structures consisting of microbial cell community surrounded by an extracellular polysaccharide substance (Pereira et al., 2021; Wall et al., 2019). Research indicates that microbial cells within biofilms are 10 to 100 times more resistant to antibiotics than planktonic cells (Sharma et al., 2019). Consistent with this, in our study, the IC_50_ of metformin for planktonic *C. albicans* was 4 mg/ml, while the IC_50_ for *C. albicans* biofilm was 64 mg/ml, 16 times that of planktonic cells, further confirming this phenomenon. Moreover, although the in vitro drug concentrations required for biofilm clearance may seem high, it is important to note that drug concentrations required for effective in vivo treatment may be lower than in vitro due to the host immune response and tissue interactions (Kher and Santoro, 2023; Vyas et al., 2022), and the used concentration in vivo might be far lower than 64 mg/ml.

The time-kill curve remains approximately horizontal when the drug has an inhibitory effect, while the slope of the curve is negative when the drug exerts a bactericidal effect (Bren et al., 2023; Zhang et al., 2023). In this experiment, when planktonic *C. albicans* was treated with 8, 16, or 32 mg/ml of metformin, the time-kill curve remained approximately horizontal and then gradually increased, indicating that metformin showed inhibitory effects on the planktonic *C. albicans*. Xu et al. (2018) showed that metformin can inhibit planktonic *C. glabrata*, which is consistent with the results of metformin on planktonic *C. albicans* in this experiment. However, at 64 mg/ml, metformin appeared to have fungicidal effects on planktonic cells, as no *C. albicans* regeneration was observed in 72 h. Moreover, the time-kill curve for *C. albicans* biofilms treated with 32, 64, or 128 mg/ml of metformin showed a negative slope. The slopes of the time-kill curves of the 128 and 256 mg/ml metformin-treated groups were significantly reduced compared to the 32 and 64 mg/ml metformin-treated groups. These results showed that metformin had a fungicidal effect on *C. albicans* biofilm in a dose-dependent manner. The results of the time-kill curves of metformin on planktonic cells and biofilms are not entirely the same due to the different biological properties of *C. albicans* in these two states, which lead to different responses to external stimuli.

The effect of metformin on the morphology of *C. albicans* was assessed using the hyphae formation assay and SEM. Results indicated a dose-dependent inhibition of hyphae formation by metformin. Treatment with 32, 64, and 128 mg/ml metformin completely inhibited hyphae formation, maintaining the yeast form. Hyphae formation in *C. albicans* is associated with virulence factors such as adhesion, biofilm formation, and invasiveness (Krysan, 2023). Thus, metformin's inhibition of hyphal growth may reduce the virulence of the fungus. Additionally, SEM analysis revealed that *C. albicans* in planktonic and biofilm treated with metformin exhibited rough surfaces, collapse, shrinkage, bulging, and other morphological changes in a dose-dependent manner.

Mitochondrial complex I inhibition is a well-established mechanism of metformin action in cells (Abrosimov et al., 2024; Yang et al., 2021). In this study, we used the JC-1 fluorescent probe to detect the MMP of *C. albicans* treated with metformin (Carrageta et al., 2022). JC-1 selectively localizes to mitochondria, where its fluorescence properties shift with changes in MMP. In healthy mitochondria with intact MMP, JC-1 forms aggregate that emit red fluorescence. When MMP decreases, JC-1 exists as monomers, emitting green fluorescence. Our findings demonstrated that after treatment with metformin, the MMP of *C. albicans* decreased and green fluorescence increased. A reduction in MMP is generally associated with a decrease in the efficiency of the electron transport chain, leading to electron leakage and the formation of more ROS (Bagkos et al., 2014; Zhao et al., 2024). We further detected ROS levels in *C. albicans* using the DCFH-DA fluorescence probe, and the results showed that the ROS levels in the metformin groups were higher than in the control group, which is consistent with the decrease in MMP. In tumor cells, the MMP changes caused by metformin through complex I inhibition are considered one of its anticancer mechanisms, as they limit the ability of tumor cells to respond to external pressures and stimuli (Catalano et al., 2023; Sun et al., 2024). Additionally, metformin has been reported to inhibit the growth of *C. elegans* by inhibiting mitochondrial respiration (Chen et al., 2017). The mitochondrial dysfunction observed in *C. albicans* in this study parallels the effects of metformin on the mitochondria of cancer cells and *C. elegans*, suggesting a conserved mechanism of action.

Antioxidant genes such as *sod1-5*, *trr1*, *cat1-2*, and *glr1* are upregulate, playing a crucial role in clearing ROS and repairing oxidative damage when ROS level in *C. albicans* increases (Feng et al., 2022; Kang and Kwak, 2021). However, this study showed that after treatment with metformin, the expression of the *sod1* gene was downregulated in both planktonic cells and biofilms, which may affect the clearance of ROS and the repair of oxidative damage in *C. albicans*. Therefore, Both the decreased MMP and the downregulation of antioxidant genes may explain the increased ROS levels in *C. albicans* after treated with metformin. In biofilm, after treatment with 64 mg/ml metformin, the expression of *trr1* was upregulated, which may be a stress response of *C. albicans* biofilm to increased ROS. After metformin treatment, the expression of *sod3*, *cat1-2*, and *glr1* in both planktonic and biofilm forms showed no significant changes, which may due to the influence of metformin on ROS clearance and oxidative damage repair in *C. albicans*.

Autophagosomes are membrane-enclosed vesicles, typically appearing as round or oval structures under TEM, with a size range of 300–900 nm. Their interior contains degraded intracellular material, which typically appears darker (Yang et al., 2022). Autophagy plays a crucial role in maintaining cellular homeostasis (Sengking and Mahakkanukrauh, 2024), and metformin exerts its pharmacological effects through autophagy (Fang et al., 2023; Gao et al., 2020; Zhao et al., 2019). In this study, we used RT-*q*PCR and TEM to examine autophagy in *C. albicans* after metformin treatment. The results showed that metformin had different effects on autophagy in planktonic and biofilm forms of *C. albicans*: autophagy was activated in planktonic *C. albicans*, whereas it was inhibited in *C. albicans* biofilm. This difference may be attributed to the baseline difference in autophagy levels between the two states. Previous studies have shown that the autophagy level in *C. albicans* biofilm is higher than in its planktonic state (Liu et al., 2022; Shen et al., 2023). In the planktonic state, metformin may lead to overactivation of autophagy, thereby affecting cell growth. In the biofilm state, due to the typically low-oxygen or even hypoxic microenvironment, metformin may inhibit the autophagy pathway, thereby affecting the survival of the cells.

In vitro studies have shown that metformin increases the susceptibility of *C. glabrata* to azole drugs (Xu et al., 2018). Therefore, we investigated the effect of metformin combined with antifungal drugs to determine whether it could reduce the effective concentration of both drugs against *C. albicans*. The FICI method, based on IC_50_ calculations, was used to evaluate the combined effects of the drugs. This method is widely recognized for its simplicity, practicality, and reliability (Li et al., 2015). In this study, metformin exhibited synergistic effects with amphotericin B against planktonic *C. albicans* and with caspofungin against *C. albicans* biofilm. These findings suggest that metformin, when combined with specific antifungal drugs, can reduce toxicity and enhance the therapeutic effects of both agents. However, metformin combined with 5-fluorocytosine showed an antagonistic effect against planktonic *C. albicans* and an indifferent effect against *C. albicans* biofilm. These results indicate that the combination of metformin with antifungal drugs should be carefully considered, and 5-fluorocytosine should be avoided in such combinations.

In conclusion, metformin exhibits anticandidal effects on both planktonic and biofilm forms of *C. albicans*, including the inhibition of hyphal formation. Its mechanisms involve reducing MMP, increasing ROS levels, and modulating autophagy. Additionally, metformin demonstrates synergistic effects when combined with amphotericin B against planktonic *C. albicans* and with caspofungin against *C. albicans* biofilm.

## Figures and Tables

**Table 1.** Primer sequences of ROS related genes used in the present study

Genes	Sequence (5’→3’)
*trr1*	F: GACCAACTCAAGACCGACGAAGC
	R: GCCATACATCCACTACCAGCAGAAG
*sod1*	F: ACAAGAATCCGAATCCGCTCCAAC
	R: AGGACCAGCAGAAGTACAACCATTG
*sod2*	F: GAACAAGCCGTTGAAGCCAA
	R: ACCTTGAGAGACAGGAGCCA
*sod3*	F: CCAAGGATCAGGTTGGGCAT
	R: AGTACGCATGTTCCCAAGCA
*sod4*	F: ACGATACTGCAAGTGCTGCT
	R: CAGCACCGCTACCTTGAGAA
*sod5*	F: ACTTTGCTTGACGAGGGACA
	R: CAGCGCCATTACCTTGAGGA
*cat1*	F: CCAATTCCAGAACCATTTGCCACTC
	R: ACCATAAGCACCGGAACCTTTAGC
*cat2*	F: TCAAGAATGGACGCCACACC
	R: TTCTTCATCGTGGGCAGCAA
*glr1*	F: TGACAAGACTTTGATCGCCACTGG
	R: TCCAAGGCAAAGAACCCATCAGATG
*β-actin*	F: GACCAAGAAGACATCAAGGTATCAT
	R: GTGTTCAATTGGGTATCTCAAG

**Table 2.** Primer sequences of autophagy related genes used in the present study

Genes	Sequence (5’→3’)
*atg1*	F: TACAACCCAACTGAGCGGAT
	R: GTAGTGGGTGATGGGCTTCT
*atg2*	F: GCCAAGACTACGGGGTATGA
	R: GCCAAGACTACGGGGTATGA
*atg3*	F: AACGTGGCAATGGGGTAATG
	R: CGTCCTCCTCTTCCTCTTCC
*atg4*	F: AGTGGTAGAGACGCCAATCA
	R: GCACCGGTAATGTATGTGGG
*atg5*	F: CATGACTTGGGTTGCTGGAC
	R: TGTTGGCTTCACTCAATTGCA
*atg6*	F: GCCAGAAATCAATGCCGCAT
	R: CCATCTACTGCATCCTTGGC
*atg7*	F: CTGGGGTGTCAGGAGCATTA
	R: GCATCTACACCGGGGAAAAC
*atg8*	F: GCCAGAATTGCTCAGAGG
	R: GATTAATGCAGCGGTTGG
*atg9*	F: TACCGCAACACCAACAACAA
	R: CTCCGTTTCAATTGGGGTGG
*atg10*	F: GAACGTTGGCAGTTGGGATT
	R: AACCCGTATAGCCCAAACCA
*atg12*	F: ACAAGACCCAATCCCAACCA
	R: TGGTTGAATCGACGGTGTTG
*atg13*	F: AGTGTCCCGTCGTCTTCA
	R: ATGGAATCCTCATGACCCG
*atg16*	F: CCTTTTGGGACATCATTCTTCG
	R: GCGCATCGAAGACATACGA
*atg17*	F: CCATCGGAGTTCAAGCTTCC
	R: TCCGGTGATCATGTCCATCA
*atg27*	F: GCCACCTTCGCAAACAAA
	R: TGAAACCAAGCACCACCA
*ccz1*	F: TGTCTCCCAAAGCCGAATCT
	R: GTTGTTGTGGACGTGGTTGA

**Table 3.** The interactions between metformin and antifungals on *C. albicans* using the FICI model

Drug combination with metformin	Planktonic		Biofilm	
	FICI	Interpretation	FICI	Interpretation
Fluconazole	2	IND	1	ADD
Itraconazole	0.75	ADD	0.75	ADD
Amphotericin B	0.375	SYN	1	ADD
Caspofungin	1.5	IND	0.125	SYN
5-Fluorocytosine	3	ANT	1.5	IND
Terbinafine	1.125	IND	1.25	IND

SYN: synergism; ADD: additivity; IND: indifference; ANT: antagonism
